# Electrophysiological Evidence for Distinct Proactive Control Mechanisms in a Stop-Signal Task: An Individual Differences Approach

**DOI:** 10.3389/fpsyg.2020.01105

**Published:** 2020-05-27

**Authors:** Woo-Tek Lee, Min-Suk Kang

**Affiliations:** ^1^Department of Psychology, Sungkyunkwan University, Seoul, South Korea; ^2^Center for Neuroscience and Imaging Research, Institute for Basic Science, Suwon, South Korea

**Keywords:** proactive control, stop-signal task, ERP, individual differences, inhibitory control

## Abstract

Proactive control reflects a sustained, top-down maintenance of a goal representation prior to task-related events, whereas reactive control reflects a transient, bottom-up goal reactivation in response to them. We designed a manual stop-signal task to isolate electrophysiological signals specifically involved in proactive control. Participants performed a simple choice reaction time task but had to withhold their response to an infrequent stop signal, resulting in go- and stop-signal trials. We manipulated the stop-signal probability (30% vs. 10%) over different blocks of trials so that different proactive control levels were sustained within each block. The behavioral results indicated that most participants proactively changed their behaviors. The reaction times in the go trials increased and the number of response errors in the stop-signal trials decreased. However, those two behavioral measures did not correlate: individuals with an increased delayed reaction did not necessarily manifest a higher decrease in response errors in the stop-signal trials. To isolate the proactive control signal, we obtained event-related potentials (ERPs) locked to an uninformative fixation onset and compared the signals between the two stop-signal probability conditions. We found that the ERPs at the left hemisphere were more negatively shifted with the increasing stop-signal probability. Moreover, ERP differences obtained from a set of electrodes in the left hemisphere accounted for the changes in response errors in the stop-signal trials but did not explain the changes in reaction times of the go trials. Together, the behavioral and electrophysiological results suggest that proactive control mechanisms reducing erroneous responses of the stop-signal trials are different from mechanisms slowing reaction times of the go trials.

## Introduction

Cognitive control regulates our thoughts and actions according to behavioral goals upon an event and contextual information (Miller and Cohen, 2001). Braver (2012) proposed dual mechanisms of control in which reactive control refers to a transient, stimulus-driven goal activation, whereas proactive control refers to a sustained and anticipatory goal maintenance. One particular example of the dual mechanism is the inhibitory motor control; driving near a school zone provides such an example. We quickly brake the car in response to an event, such as seeing a child running onto the road to catch a soccer ball (reactive control). However, we also slow the car down and are more vigilant around any children in response to the contextual information, such as a warning sign of children crossing near a school zone (proactive control).

In laboratories, the inhibitory motor control has been studied by using stop-signal tasks (Logan, 1994). In the stop-signal tasks, participants perform a simple choice reaction time task, but they need to countermand a prepotent response upon a stop signal. If the stop signal occurs with a delay, participants tend to respond despite the stop signal. In contrast, if the signal occurs immediately after a target to respond, participants tend to withhold the responses. This systematic relation between the response tendency and the stop-signal delay in conjunction with race models offers a means to calculate stop-signal reaction times (SSRTs), a measure to infer unobservable inhibitory mechanisms (Logan and Cowan, 1984; Band and van Boxtel, 1999). Stop-signal tasks powered with SSRTs have been a popular behavioral protocol for studying inhibitory mechanisms in normal participants and in patients with various psychiatric disorders (Verbruggen and Logan, 2008; Verbruggen et al., 2019).

Herein, we investigated the electrophysiological signature of proactive control in response inhibition with a stop-signal task. Several experimental variables influence proactive control in the stop-signal tasks. For example, knowledge of the upcoming trial type and relevant stimulus dimension modulate the response inhibition and attentional setting (Elchlepp et al., 2016). A recent addition to the literature is reward, which an experimenter can use to manipulate the motivational level of participants (Greenhouse and Wessel, 2013; Schevernels et al., 2015). Nevertheless, one dominant way to manipulate the anticipatory contextual information is the stop-signal probability, either with a number (van Belle et al., 2014; Vink et al., 2015) or with a semantic cue such as “maybe” (Verbruggen and Logan, 2009; Swann et al., 2013). By increasing the frequency of stop signals, reaction times are slowed down, accuracy for the choice task increases in the go trials, and responses tend to be inhibited in the stop-signal trials, however, decreasing the frequency results in opposite effects. The stop-signal probability manipulation is so powerful that even short-term changes in the stop-signal frequency lead to behavioral adjustments (Mirabella et al., 2006; Emeric et al., 2007).

There are several reasons to study electrophysiological signals carrying proactive control in the response inhibition. Electrophysiological studies on motor execution have shown that prior information for action and, thus, more preparation increase the negativities (Leuthold et al., 2004). Specifically, the contingent negative variation (CNV) is a negativity that accumulates with anticipation of a target (Walter et al., 1964; Brunia, 2003) and is a potential candidate of proactive control variables as it reflects a preparation of stimulus-response mappings (Verleger et al., 2000; Leuthold et al., 2004; Brunia et al., 2010; Kang et al., 2014). Accordingly, several studies have shown negative modulations in relation to the proactive control in stop-signal tasks (Schevernels et al., 2015; Elchlepp et al., 2016; Elchlepp and Verbruggen, 2017; Liebrand et al., 2017; Steinhauser and Steinhauser, 2018) by analyzing electrophysiological signals following informative cues, targets of choice tasks, and stop-signals. However, we reasoned that the procedure is problematic because proactive control signals can be buried by signals processing sensory as well as motor processes.

Here, we sought to identify electrophysiological signals of proactive control variables that accompany the stop-signal tasks with the following strategies. First, we manipulated the stop-signal probability over different blocks of trials so that proactive control variables were sustained within each block. It is possible to provide a foreknowledge of the stop-signal probability in a trial-by-trial basis. However, many studies on task switching have pointed out that our ability to voluntarily switch tasks based on a foreknowledge is limited (Kiesel et al., 2010) and that neural signals associated with cue processing can be more pronounced than proactive control signals. It is, therefore, reasonable to separate the two conditions over different blocks of trials to eliminate cue-related processes. Second, we measured electrophysiological responses locked to the uninformative fixation onset to minimize the contribution of the sensory and motor processes. The fixation onset is an attractive choice because it does not inform participants about target type or the occurrence of the stop-signal, while providing an event for locking electrophysiological signals, especially ERPs (Event-Related Potentials). Finally, we adopted an individual differences approach with various behaviors obtained from the stop-signal tasks with a rationale that if the obtained ERPs carry a proactive control variable, the signal should explain behavior.

We hypothesized that the increase of the stop-signal probability should elevate the proactive control levels. As a result, reaction times of the go trials should become slower and erroneous responses of the stop-signal trials should increase in the 30% stop-signal probability condition than the 10% condition. In addition, if the CNV does carry proactive control variables, the negativity should increase in a sustained manner with increasing stop-signal probability and the modulation should explain the behavior changes between the two probability conditions as well.

## Materials and Methods

### Participants

Thirty-eight participants (16 females; mean age, 23.8 years; range, 19–27) provided informed consent to this study approved by the Sungkyunkwan University’s institutional review board prior to participating. Participants declared having normal color vision and visual acuity and no neurological diseases. Each participant received a monetary compensation of 20,000 KRW (approximately 20 USD) per hour.

We planned to analyze approximately 30 participants’ data and determined to collect data sets from 36 participants with following reasons. We set 0.8 for our target power and 0.45 for a target correlation with 0.05 alpha level, resulting in 29 participants. To determine this number in the absence of any prior knowledge of the electrophysiological signals associated with the proactive control variables, we consulted previous studies on CDA (Contrlateral Delay Activity). CDA is an electrophysiological index of visual working memory and it is well established the CDA correlated with a filtering efficiency, how well we can filter out task-irrelevant stimuli when encoding items in visual working memory. We focused on the filtering efficiency rather than capacity because a high and robust correlation between CDA and capacity measure can be taxing for an exploratory nature of the present study. Luria et al. (2016) compiled 9 experiments from 200 participants (approximately 22.2 participants/experiment) and showed that the average correlation was 0.478, resulting in a power of 0.77. We increased the target power from 0.77 to 0.8 and assumed that correlation can be worse than 0.478. We therefore planned to analyze approximately 30 participants and added approximately 20% more participants for potential loss of data sets from poor recording quality and behavioral performances.

We included 33 participants’ data for behavior analyses and 27 participants’ data for the ERP analyses. Specifically, three participants did not complete the study. Data of six participants were excluded from the ERP analyses due to excessive noise in signals, including oculomotor artifacts and signal fluctuations, resulting in trial loss greater than 50% (mean = 62.9%, range 51.8–80.2%). While we applied independent component analysis (ICA) for removing eye movements, <50% noise-free trials prior to the ICA application were concerning (see section EEG Acquisition and Analysis. for detailed rejection procedure). We further excluded data of two participants based on their behavioral performances, due to a possible waiting strategy (Logan, 1994), which we describe in detail in Results section.

### Apparatus

The experiment was performed on a Mac-mini running Matlab with a psychophysics toolbox (Brainard, 1997; Pelli, 1997). All visual stimuli were presented on a 21-in CRT monitor (100 Hz in refresh rate, 1024 × 768 in resolution) placed approximately 70 cm away from the participants.

### Stimuli and Procedure

**FIGURE 1:**
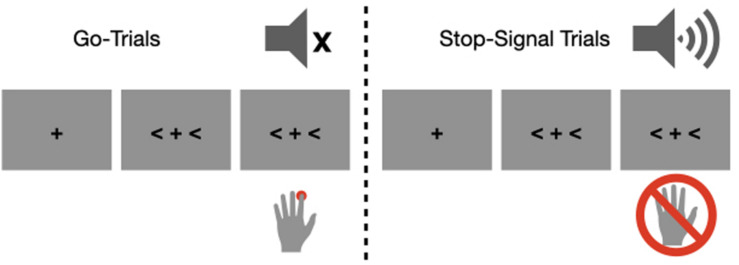
An illustration of the stimulus sequence. In the go trials (left), participants needed to respond to the direction of the arrow signs. In the stop-signal trials (right), participants needed to countermand the action in response to the auditory stop signal.

The participants performed a manual stop-signal task (Figure 1). They initiated each block of trials by pressing the spacebar. A trial consisted of a fixation point and a target stimulus, which was presented at the center of the computer screen. We varied the interval between the fixation point and the target to control a fixed temporal expectation (an equal number of 500 and 1000 ms trials for the short and long fore-period trials, respectively) even though we planned to analyze slow potentials only from the 1000 ms trials to reduce overlap between sensory (i.e., processing fixation) and motor processes. In retrospect, nevertheless, we acknowledged that randomly jittering fore-period (e.g., 600–1400 ms) would have been more efficient to control temporal expectation as well as to prevent overlap between sensory and motor processes. The target stimuli were either left- or right-pointing arrows occupying both sides of the fixation point, and participants needed to indicate the direction of the arrow by pressing the “F” button (left arrow) or the “J” button (right arrow) with the index finger of the left and right hand. Participants were instructed to respond as quickly and accurately as possible to the go stimulus, but they were also instructed to countermand their response as best as they can when the stop signal was presented. We nevertheless emphasized participants’ responding to the arrow target in order to discourage any waiting strategies by informing that it is natural to make an erroneous response in stop-signal trials. Note that we did not provide any feedback for especially slow responses, which would have been more efficient to prevent any waiting strategies. The stop-signal was a 1000 Hz auditory tone lasting 100 ms (65 db). To manipulate the anticipatory context, the stop-signal probability (10% vs. 30%) was varied over different blocks of trials, and participants were informed of the stop-signal probability of a given block at the beginning of each block. The 10- and 30%-probability blocks were repeated 20 times in a pseudo random order and each block consisted of 24 trials. As a result, each participant completed 960 trials distributed over 40 blocks.

We decided to use the proportion of response errors as a behavioral measure of the stop-signal trials, while fixing stop-signal delays (SSDs) across all participants. We determined three SSDs (150, 250, or 350 ms) based on a pilot study and used them for all participants. Using a fixed SSD procedure, rather than a tracking procedure, is not a common practice in stop-signal literature because the tracking procedure is more suitable for calculating SSRTs (Verbruggen et al., 2019). Nevertheless, we decided to use the same SSDs for all participants for the following reasons. First, in the tracking procedure, SSD increased when participants made a response in the stop-signal trial but decreased when they successfully inhibited their action by approximately 50 ms. If the tracking procedure was used, all participants responded with a similar proportion of trials in the stop-signal trial (approximately 50%). Although it is possible to estimate an individual participant’s SSD (e.g., mean) as a behavioral index, we were concerned that the measure could be insensitive to an individual differences approach. Second, if we adopted a tracking procedure, SSDs can vary across the two stop-signal probability blocks. In addition, with a similar level of performance between the two blocks, this could equate their anticipation level. Third, nevertheless, because one of those three SSDs was randomly selected in a given stops signal trial, participants cannot predict when the stop signal occurs.

### EEG Acquisition and Analysis

EEG data were recorded at 500 Hz using 32 Ag/AgCl electrodes. Twenty-eight electrodes were placed according to the international 10-20 system (Fp1, Fp2, F3, F4, F7, F8, Fz, FC1, FC2, FC5, FC6, C3, C4, Cz, CP1, CP2, CP5, CP6, P3, P4, P7, P8, Pz, O1, O2, Oz, T7, and T8). EEG was referenced online using the left mastoid, while the right mastoid signals were recorded. The electrodes were re-referenced offline to the average of the right and left mastoids (Nunez and Srinivasan, 2006). Horizontal eye movement was monitored using the horizontal electrooculography (EOG) channel located at the external canthus of the right eye, and the vertical EOG channel was placed approximately 3 cm below the left eye. Both EOG channels were referenced to the left mastoid as well. All data were bandpass filtered online, from 0.01 to 100 Hz.

The ERP waveforms were time-locked to the onset of fixation point and the baseline was corrected to an interval of 200–0 ms before its onset. Waveforms were low-pass filtered (a two-way least-squares finite-impulse- response filter with 0.01 and 40 Hz for low and high end of the frequency band). Fp1 and Fp2 electrodes tended to show severe noise and, thus, we did not include those two electrodes in the entire analyses. We initially determined the noisy data set based on a conventional epoch-based rejection (Luck, 2005). We then applied independent component analysis (ICA) to the remaining data set to remove eye movement-related artifacts (Makeig et al., 1996) and identified trials with other types of noises (e.g., slow drift) between a −200 ms and 1000 ms window with the same epoch-based rejection procedure (Luck, 2005), resulting in 11.2% trial loss (0.2–28.8%). This additional rejection includes trials with signal drifts, muscle noises and abnormal signal fluctuations at least one of the channels. Note that we did not include the first trial of each block for the analysis and performed the entire analysis with and without ICA, and the results were comparable.

Because it was novel to look into proactive signals locked with uninformative fixation point, it was difficult to set *a priori* hypothesis to test. We therefore conducted two types of analyses that complemented each other guided by Luck and Gaspelin (2017). In one, we took a mass univariate approach based on a cluster-based permutation test for every time point (−0.2 to 1.0 s) and electrode, and the multiple comparison problem was addressed by a cluster-based correction, implemented by Fieldtrip toolbox (Oostenveld et al., 2011). We used the *triangulation* method to build a list of neighboring electrodes and randomized the sample 10,000 times. Because we hypothesized a greater negativity with increasing stop-signal probability, we conducted a one-tailed t test with alpha equaling 0.05. Cluster size was used for the cluster statistics with its alpha equaling 0.05 and minimum number of electrodes equaling 2. In the other analysis, we applied a collapsed localizer approach, in which we averaged ERPs from both low and high probability conditions and identified a set of electrodes and temporal window. We then conducted a 3-way ANOVA with factors of probability (low and high) and two electrode positions (anterior, central and posterior X left, medial and right).

## Results

### Behavioral Evidence for Proactive Control

The behavioral results indicated that participants proactively adjusted their behaviors. Table 1 summarizes behaviors of reaction times, accuracy, and omission errors of the go trials and response errors of the stop-signal trials for the two fore-periods and two stop-signal probability conditions. These results are consistent with the previous findings. Note that we did not analyze SSRTs because our fixed SSD procedure is not suitable for computing SSRTs (Verbruggen et al., 2019). Instead of analyzing these behavioral patterns that are already well established, we focused on individual behaviors associated with the long fore-period trials below. The reason we focused on the long fore-period trials was because the target was presented 500 ms after the fixation onset of the short fore-period trials and, thus, it was difficult to identify any sustained proactive control variables with the fixation-lock ERPs. In addition, we did not analyze the accuracy of the go trials because the accuracy was close to the ceiling.

**TABLE 1:** Behavioral results are presented as the mean (standard deviation).

**Stop-signal probability**	**Stop-signal trial**		**Go trial**					
	**Erroneous response (%)**		**Reaction times (ms)**		**Accuracy (%)**		**Omission error (%)**	
	**Short**	**Long**	**Short**	**Long**	**Short**	**Long**	**Short**	**Long**
10%	51.02 (18.63)	53.44 (19.48)	437.0 (52.0)	433.4 (50.8)	98.93 (1.99)	98.64 (2.27)	0.064 (0.20)	0.032 (0.12)
30%	43.79 (13.31)	46.36 (14.40)	460.6 (53.7)	454.7 (51.1)	99.22 (1.58)	99.08 (1.96)	0.060 (0.18)	0.041 (0.16)

**FIGURE 2:**
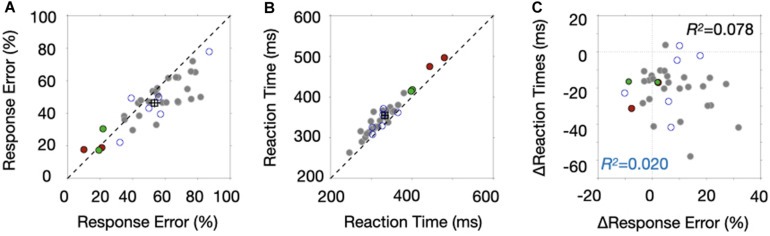
Behavioral results. **(A)** The proportion of response errors of the high probability block is plotted as a function of response errors of the low probability block. Each data point corresponds to the mean response probability of each participant. The square marker is the average, and the error bars indicate ± SEM (standard error of the mean). The red filled circles and blue open circles indicate data points excluded from the ERP analysis due to their waiting strategy and noisy ERPs, respectively. The green boundary circles were included in the analysis. **(B)** Response times of the high probability block are plotted as a function of response times of the low probability block. Other aspects are the same as those in Figure 2A. **(C)** Reaction time difference between the two probability conditions is plotted as a function of the difference in response errors of the stop-signal trials.

We presented the results such that we could highlight differences between the high- and low stop-signal probability conditions (low- and high probability condition afterward) across individuals. Figure 2A shows the stop-signal performance between the low- and high probability conditions of all 35 participants who completed the experiment. The stop-signal performance was summarized such that the proportion of response errors across the three SSDs was averaged and the performance of the high probability condition was plotted as a function of the performance of the low probability condition. We included the two green data points despite their low response probability, but their exclusion did not change the results. Two red data points were excluded for further analyses because we reasoned that these participants adopted a waiting strategy with slow reaction times, discussed next. We also excluded six participants’ data marked with blue open circles for the ERP analyses because of their noisy ERP signals. The empty square box represents the mean of the data points with error bars after excluding two red and six open blue circle data.

We found that the response probability of the stop-signal trials was lower in the high probability condition than in the low probability condition [*t*(26) = 3.9338, *p* = 0.0005]. In addition, the data points lay below the dotted unity line with increasing response errors. Response times were slower in the high probability condition than in the low probability condition [*t*(26) = −8.7724, *p* < 0.0001] (Figure 2B). Note that reaction times of those two data points marked with red were much slower than those of other points, confirming our suspicion that participants adopted a waiting strategy. Surprisingly, we found that the differences of the two behavioral measures were not correlated (Figure 2C), whether we included the six data sets associated with noisy ERPs (*R*^2^ = 0.020, *p* = 0.4112, Bayse Factor, BF10 = 0.4202) or not (*R*^2^ = 0.078, *p* = 0.1413, BF10 = 0.1836). This lack of correlation suggests that at least two different proactive control mechanisms are operating in stop-signal tasks and provide a reason to correlate ERP modulations with those two behaviors separately.

To the best of our knowledge, the lack of correlation has not been documented in the literature. We reasoned that our fixed SSD procedure provided an opportunity to reveal the importance of stop-signal performance (see Materials and Methods section for our justification of using a fixed SSD procedure).

### Lateralized ERPs Explain Behavior Changes in Stop-Signal Trials

**FIGURE 3:**
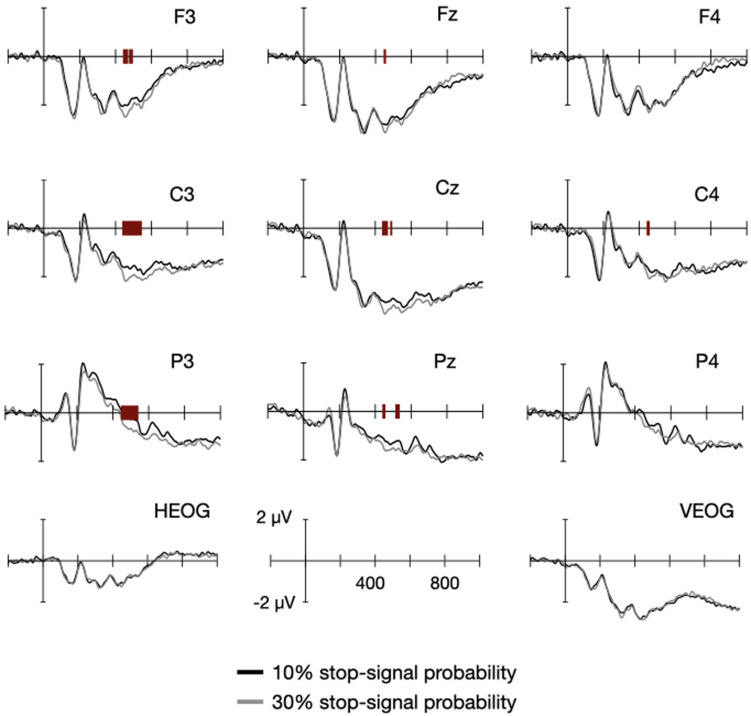
Event-related potentials (ERPs) between the high- and low probability conditions. ERPs were locked to the onset of fixation for the nine electrodes around Cz. The black and gray lines are associated with the low- and high probability conditions, respectively. The red bars at the x-axis indicate the statistically significant difference between the two conditions of the corresponding time point.

Figure 3 shows the fixation-locked ERPs from the go trials, in which the black and gray lines indicate ERPs from the low- and high probability conditions, respectively. The red bars at the x-axis indicate the significant difference at 0.05 alpha level, identified from a cluster-based permutation test, which is described below. The negativity was broadly distributed the frontocentral area at an earlier phase, and it developed slowly and sustained at the centroparietal area at a later phase of the fixation interval.

Guided by Luck and Gaspelin (2017), we analyzed the entire data set with two different approaches that could complement each other. One is the mass univariate approach and the other is the collapsed localizers. While most of previous studies analyzed various electrophysiological signals locked with the onset of informative stimulus, we planned to analyze ERPs locked with uninformative fixation point and, thus, it was difficult to set a specific *a priori* hypothesis to test. We therefore took those two approaches that can complement each other.

#### Mass Univariate Approach

**FIGURE 4:**
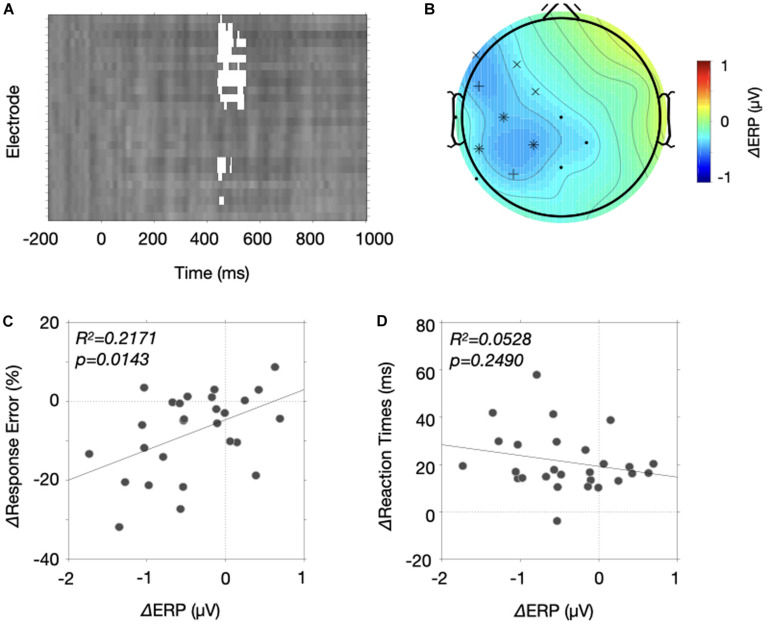
Results of the mass univariate approach. **(A)** A map of significant differences (white boxes) is drawn for each electrode as a function of time. The 26 electrodes were arranged from the top to bottom in order of Fz, F3, F7, FC5, FC1, C3, T7, CP5, CP1, Pz, P3, P7, O1, Oz, O2, P4, P8, CP6, CP2, Cz, C4, T8, FC6, FC2, F4, and F8. The brightness changes indicate the ERP differences between the two probability conditions. **(B)** The temporal extent of the significant differences of an electrode was plotted on its position. We only selected electrode whose significance differences longer than 20 ms throughout 0.2–1.0 s (* for ≥100 ms, + for ≥80 ms, x for ≥40 ms, • for ≥20 ms). The ERP differences between 450 and 550 ms were color coded to aid the interpretation. **(C,D)** Correlation between the ERP differences and the behavioral differences. **(C)** The response error differences of the stop-signal trials were plotted as a function of ERP differences between the two probability conditions. The solid line indicates a fitted line. **(D)** The reaction time differences of the stop-signal trials were plotted as a function of ERP differences between the two probability conditions. The solid line indicates a fitted line.

We first identified any ERP modulations between the high- and low probability conditions with the 27 participants’ data. The data points at every time between −0.2 and 1.0 s and electrode were submitted to a cluster-based permutation test in which the number of false positive findings were controlled with a cluster-based correction. We represented significant differences between the two probability conditions for a subset of electrodes with red bars shown in Figure 3 and for the entire electrodes with a two-dimensional map shown in Figure 4A. The brightness in gray scale indicates the ERP difference at a given electrode and time pair, and the white boxes indicate the samples showing significant effect of the stop-signal probability. It is easy to see that the significant differences were limited within a short temporal window (442 and 546 ms), but difficult to imagine that those separated boxes actually belong to a single cluster. We therefore create a topography (Figure 4B) in which the number of time points showing significant probability effect was summarized by the markers if it is greater than 20 ms (^∗^ for ≥100 ms, + for ≥80 ms, x for ≥40 ms, • for ≥20 ms) on top of the color coded average voltage differences between 450 and 550 ms. As evident from the figure, significant differences were broadly distributed in the left hemisphere and lasted for a longer period of time for the electrodes close to centroparietal area. With a sizeable variability in response errors of the stop-signal trials and reaction times of the go trials, we adopted an individual differences approach to identify proactive variables carried in this lateralized negativity in the next.

If the significant ERP differences do carry proactive control variables, they should explain behavior differences across individuals. We analyzed the two types of behavioral changes separately because we have shown that response errors of the stop-signal trials decreased and reaction times of the go trials increased with stop signal probability, and those two measures were uncorrelated. We plotted the behavioral changes of the response errors of the stop-signal trials (Figure 4C) and the reaction times of the go trials (Figure 4D) as a function of the ERP differences between the two probability conditions. Because we hypothesized that signals in relation to proactive control were sustained, we averaged the ERP differences from the three centroparietal electrodes (C3, C5, CP1) in which significant effects lasted long (>100 ms). We found that the ERP differences explained the changes in response errors of the stop-signal trials

(*R*^2^ = 0.2171, *p* = 0.0143), but they could not explain the changes in reaction times (*R*^2^ = 0.0528, *p* = 0.2490). These results indicate that the lateralized, mostly centroparietal area, negativity carries a proactive control variable that facilitates stopping behaviors of the stop-signal trials.

However, the cluster-based permutation test may be limited for our study because we reasoned that the proactive control variables should be sustained during the fixation interval. Specifically, in general, we acknowledge that the cluster-based non-parametric approach is suitable for controlling the false positive findings in electrophysiological studies because it considers the spatiotemporal profile of the signal. Conceptually, two significant differences in the absence of any true signal can occur in any two electrode and at any two time points, but the two significant differences should be clustered between adjacent electrodes or time points if they reflect a true effect originated from a common source. However, if the number of electrodes is small and, thus, the distance between any two adjacent electrodes become large, the cluster-based permutation test is not suitable for identifying a very localized effect. In addition, more importantly, the results of the cluster-based permutation test depend on the threshold for selecting samples for the cluster, the clusteralpha parameter of the fieldtrip package. As noted by Maris and Oostenveld (2007), “for a weak and widespread effect, the threshold should be low, and for a strong and localized effect, the threshold should be high” (p. 189). Out of these two concerns, if we decreased the minimum number of channels contributing the cluster or decreased the selection threshold for the cluster, we obtained multiple clusters lasting longer period of time, seemingly consistent with our expectation of sustained modulation. Nevertheless, the selection procedure became increasingly arbitrary and, thus, we reanalyzed the entire data set based on the procedure termed collapsed localizers by Luck and Gaspelin (2017).

#### Collapsed Localizers Approach

CNV is a candidate of ERP component of proactive control variables (Verleger et al., 2000; Leuthold et al., 2004; Brunia et al., 2010; Kang et al., 2014), which shows a broad negativity around the Cz electrode. We hypothesized that the increased proactive control variables accompanied with the stop-signal probability elicited a greater negativity with a three-way ANOVA with factors of stop-signal probability (low and high), two factors of electrode position (anterior, central and posterior X left, medial, and right). This is because we did not expect that the CNV modulation should be comparable between the two hemispheres or between the anterior and posterior areas. Specifically, while it is true that the modulation is usually maximal at the Cz electrode, both frontocentral as well centroparietal modulations have been discussed with different functional roles (Bender et al., 2004; Leuthold et al., 2004). In addition, task requirements (e.g., verbal vs. spatial) modulated CNVs differentially between the two hemispheres (Rebert and Lowe, 1984; Howard et al., 1992). Similarly, the topographical distribution of CNV varies anterior to posterior axis (Elchlepp et al., 2016; Grane et al., 2016; Elchlepp and Verbruggen, 2017) and it was slightly shifted toward the left (Liebrand et al., 2017).

**FIGURE 5:**
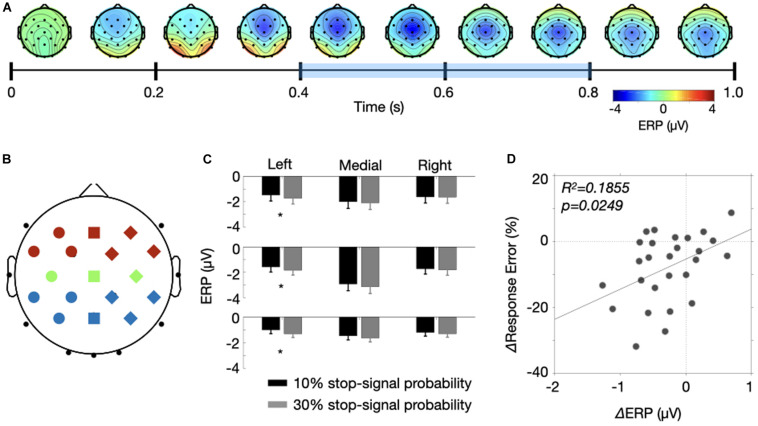
Results of the collapsed localizers approach. **(A)** A time course of CNV modulations is represented by a series of topographies. Each topography was produced by averaging two probability conditions for 100 ms starting from 0 ms with 100 ms step size. The bluish color drawn over the time axis is the temporal window of interest. **(B)** A illustration of electrode assignment for 9 positions (anterior = red, central = green, posterior = blue X left = circle, medial = square, right = diamond). **(C)** ERP amplitudes for the two probability conditions were drawn for the nine electrode positions. Error bars were ±1 S.E.M. **(D)** The response error differences of the stop-signal trials were plotted as a function of ERP differences between the two probability conditions. The solid line indicates a fitted line.

Here, we selected 17 for nine sets of electrodes (anterior, central, posterior X left, medial, right) and determined a temporal window (400–800 ms) to analyze a sustained CNV modulation between the two probability conditions. Specifically, we selected the electrode set and the temporal window after averaging ERPs of the high and low probability conditions following the collapsed localizers approach (Luck and Gaspelin, 2017). Figure 5A shows topographies of averaged ERPs over time. We averaged the ERPs over 100 ms window at every 100 ms step size from the fixation onset, resulting in 10 topographies. Like previous studies, the negative modulation was maximal at Cz and the modulation gradually changed from frontocentral areas to centroparietal areas. To include those frontocentral and centroparietal negativity, we selected those 17 electrodes, resulting in 3 × 3 electrode configurations shown in Figure 5B. Three colors represent the anterior (red), central (green) and posterior (blue) electrodes and three shapes represent the left (circles), medial (squares) and right (diamonds) electrodes. In addition, we determined a sustained temporal window from 400 to 800 ms. There are reasons for this selection. While the frontocentral modulation appears emerge from 300 ms, we excluded ERPs from 300 to 400 ms within a concern that the stimulus processing associated with fixation onset (e.g., P3, Polich, 2007). We also excluded the last 200 ms because the frontal modulations were nearly absent, and the negativity extended to occipital electrodes. Note, however, we found a similar result even when we include the last 200 ms for the analysis.

Figure 5C shows the ERP amplitudes for the nine sets of electrodes averaged across 400 to 800 ms temporal window. The negative modulation was at its maximum at Cz, but the ERPs were comparable between the two probability conditions across the electrodes except the electrodes in the left hemisphere. A three-way ANOVA with factors of stop-signal probability (low vs. high), two electrode positions (anterior, central, posterior X left, medial, right) yielded significant main effects of the two electrode positions [*F*(2, 52) = 5.828, *p* = 0.0052 for the anterior to posterior position; *F*(2, 52) = 22.78, *p* < 0.0001 for the left and right position] and their interactions [*F*(4, 104) = 13.09, *p* < 0.0001], indicating the strongest modulation at the Cz electrode. More important, we found that the stop-signal probability interacted with lateral electrode position [*F*(2, 52) = 4.694, *p* = 0.0134]. If we ran a one-way ANOVAs for the subset of electrode (left, medial, and right), we found that the stop-signal probability significantly modulated the ERPs at the left electrodes [*F*(1, 26) = 8.345, *p* = 0.0077] but not the others [*F*(1, 26) > 1.786, *p* > 0.193]. In addition, the ERP modulations obtained from those seven left electrodes explained the decreased response errors of the stop-signal trials (Figure 5D, *R*^2^ = 0.1855, *p* = 0.0249), but could not explained the response time slowing (*R*^2^ = 0.0525, *p* = 0.2504). Together, these results provide evidence that the negative potential in the left hemisphere carries proactive control variables involved in reduction of response errors of the stop-signal trials.

## Discussion

To investigate the electrophysiological signature of proactive control, we set up a novel research strategy such that we manipulated the stop-signal probability over different blocks of trials, obtained ERPs locked with uninformative fixation onset to minimize the impact of sensory and motor processes, and adopted an individual differences approach. Consistent with previous studies, participants tended to slow down reaction times in the go trials and were more likely to countermand their action in the stop-signal trials. However, those two types of behaviors were not correlated across individuals. In addition, we found that the stop-signal performance explained by the ERPs obtained from left hemisphere from two analyses, indicating that the negativity of the left hemisphere carries proactive control variables involved in reduction of response errors in the infrequent stop-signal trials.

### What do the Reduced Response Errors Reflect?

We found that the response slowing of the go trials does not correlate with reduced response errors of the stop signal trials across participants. While it is true that the additional slowing could decrease the erroneous responses of the stop-signal trials, why those two measures were uncorrelated remains to be explained.

We reasoned that subjective anticipation of the stop signal at a given trial can provide an answer. While the stop signal probability was varied over different blocks of trials, evidence indicates participants evaluate the likelihood of stop signal from trial to trial as well. When participants were given a stop signal probability at the beginning of each trials and then asked whether they expected the stop signal, response times of the go trials were slowed down with increasing probability but they were slowed down even further if they expected a stop signal (Vink et al., 2015). Previous neuroscientific studies also have pointed out that subjective anticipation of stop signal might be a proactive control variable distinguished from response slowing. Specifically, a dynamic Bayesian model revealed that we update the likelihood of stop signal based on stimulus history (Yu et al., 2009) and has been used to constrain functional roles of neural responses (Ide et al., 2013; Chang et al., 2014). With the dynamic Bayesian modeling, the anticipation of the stop signal and the response time of the go trial were mapped over different brain regions (Hu et al., 2015) and Chang et al. (2017) found that those two behavioral indices were mapped over different oscillatory rhythms. Considering that the occurrence of the stop signal trials is independent from the trial-by-trial anticipation of the stop signal, it is more reasonable to expect that those two measures are independent.

### Functional Roles of Lateralized ERPs in Proactive Control of Stop Signal Task

It is true that various electrophysiological signals such as alpha- (Bender et al., 2004) and beta-band oscillations (Liebrand et al., 2017) were clearly lateralized in the left during preparation, CNV modulation is bilateral in majority of studies (see a review by Leuthold et al., 2004) including studies of stop-signal tasks (Schevernels et al., 2015; Elchlepp et al., 2016; Elchlepp and Verbruggen, 2017; Liebrand et al., 2017; Steinhauser and Steinhauser, 2018). On the other hand, we found a robust lateralized modulation that even explains the reduced response errors of the stop-signal trials. We first discuss confounding factors that could have induced the lateralized CNV responses and then interpret the lateralized negativity in relation to anticipation.

We acknowledge that two factors could have contributed to the lateralized negative modulation of CNV. One potential confounding factor is the handedness such that the left-lateralization reflect the greater right-hand preparation accompanied with right-handed participants (Kutas et al., 1977). While we did not ask participants their handedness, the reaction time data suggested that the right-handed participants comprised a majority such that go reaction times associated with the right hand response were significantly faster than those associated with the left hand response [*t*(26) = 2.3060, *p* = 0.0293] and this pattern was observed in 18 out of 27 participants. Future studies only from the left-handed participants would provide an answer to this concern. The other confounding factor is an inherent bias in attention. Specifically, even though only the fixation point was presented during the fixation interval, which is our interval of interest, the arrow targets were presented at both side of the fixation during the target interval. The arrow stimuli may not be suitable for the go stimulus. This is because participants could have deployed their attention to one of the two that matched to their own inherent attentional bias, resulting in a negativity at the contralateral side. In addition, it is also difficult to counterbalance the response hands across participants because the pointing direction of the arrow is strongly associated with the respond hand. It means that it is difficult to respond with the right hand for the left arrow. This concern is reasonable based on previous studies showing inherent rightward bias in attention (Takio et al., 2013). Considering that sensory and motor processing are minimized during the fixation interval, these confounding factors can be more pronounced compared to other studies. Future studies with a symbolic stimulus presented at the center would provide an answer to this concern.

We nevertheless do not rule out a possibility that the left hemisphere could have played a role in reduced response errors of the stop-signal trials in relation to the “left-dominant sensorimotor network in the control of temporal attention” (Nobre and Van Ede, 2018, p. 39). In stop-signal tasks, Vink et al. (2015) identified that the BOLD response at the left premotor cortex reflected additional response slowing associated with subjective anticipation of stop signal. Jaffard et al. (2008) identified several brain areas associated with the proactive control in the left hemisphere. Left hemisphere also explained the difference between short and long SSRT groups (Li et al., 2006). Future studies controlling the confounding factors discussed above in conjunction with source localization can provide an answer whether the left hemisphere is indeed specialized for anticipating a stop-signal.

## Data Availability Statement

The datasets generated for this study are available in https://osf.io/3kt5y/.

## Ethics Statement

The studies involving human participants were reviewed and approved by Sungkyunkwan University Institutional Review Board. The patients/participants provided their written informed consent to participate in this study.

## Author Contributions

M-SK conceived the study. W-TL performed the experiment. W-TL and M-SK analyzed the data. All authors wrote and discussed the manuscript.

## Conflict of Interest

The authors declare that the research was conducted in the absence of any commercial or financial relationships that could be construed as a potential conflict of interest.
